# Extended Medial Coronal Plane Plica Formed by a Rare Fusion of Medial Patellar and Biblike Plicae in the Knee: A Case Report

**DOI:** 10.7759/cureus.59864

**Published:** 2024-05-08

**Authors:** Srinivas B. S Kambhampati, Sunil K Koneru, Riccardo D'Ambrosi, Anirudh P. S Kambhampati, Dipen K Menon

**Affiliations:** 1 Department of Orthopedics, Sri Dhaatri Orthopedic, Maternity, and Gynecology Center, Vijayawada, IND; 2 Department of Orthopedics, Pinnamaneni Siddhartha Institute of Medical Sciences and Research Foundation, Vijayawada, IND; 3 Department of CASCO, IRCCS Istituto Ortopedico Galeazzi, Milan, ITA; 4 Dipartimento di Scienze Biomediche per la Salute, Università degli Studi di MIlano, Milan, ITA; 5 Department of Orthopedics, Kettering General Hospital, NHS Foundation Trust, Kettering, GBR

**Keywords:** knee, coronal plane plica, biblike plica, case report, plica

## Abstract

The traditional plicae of the knee have been updated recently with reports of new variants in the coronal plane. We report another variant, an extended medial coronal plane plica (EMCPP), previously unreported, in a 70-year-old lady with osteoarthritis of the knee and a loose body.

The preoperative symptoms after the excision of the plica and loose body were temporarily relieved for 11 months before she underwent total knee arthroplasty (TKA) for associated osteoarthritis.

Excision of the EMCPP could resolve symptoms, but resultant internal scarring or the plica itself could cause difficulty in exposure during TKA.

## Introduction

Traditionally, four types of plicae have been described in the knee. A fifth type, the infrapatellar coronal plane plica (ICPP), has been described recently [1,2]. One of the most common symptomatic plica is the medial patellar plica. It is a coronal plane plica lying on the medial side of the knee and covers the medial femoral condyle. Various terms have been used for this plica in the literature: medial synovial shelf, Lino’s band, Aoki’s ledge, plica synovialis mediopatellaris, plica alaris elongate, and meniscus of the patella [3]. It extends from the suprapatellar pouch superiorly to the infrapatellar fat pad [3,4]. Biblike plica is a coronal plane infrapatellar plica and has been described in a series of 12 cases in one study [1]. A further report was added in a 60-year-old lady with magnetic resonance imaging (MRI) findings [2]. This plica is located entirely in the infrapatellar region.

The majority of knee synovial plica are of unknown cause, and it has been estimated that symptoms occur in both knees in up to 60% of cases, but they may not occur at the same time. Additional factors linked to trauma, excessive usage injuries, hematoma, diabetes, and inflammatory arthropathy have been discovered. During adolescence, symptoms may manifest during a period of rapid physical development. Therefore, any main condition of the knee that can cause temporary or long-lasting inflammation of the synovial membrane may be involved in the formation of an abnormal plica. The embryological explanation of synovial plica suggests that there may be a genetic factor involved with this illness. However, the specific basis for this genetic component has not been determined [5]. 

Patients may experience an exacerbation of symptoms when engaging in excessive or strenuous activities that require bending and straightening of the knee. The synovial plica is an integral element of the knee joint and is connected to the quadriceps muscles indirectly. Its position is dynamically regulated during knee flexion and extension due to its attachment to the fat pad. Plical irritation is more prevalent in people with inadequate quadriceps tone or any notable muscular imbalance around the knee. Prolonged flexion of the knee has been found to cause pain, particularly when resting at night, which can be bothersome for patients. The progression of symptoms is not a necessary outcome of the disorder, but there is yet no satisfactory resolution on how to identify patients who would undergo a gradual deterioration of symptoms without therapy [5].

We describe a plica that appears as a morphological combination of the biblike plica and the medial patellar plica, extending into the medial suprapatellar region causing symptoms. Such a structure was not previously reported in the literature. We termed it extended medial coronal plane plica (EMCPP).

## Case presentation

A 70-year-old lady presented with bilateral knee pains for the last 15 years, with increasing left-sided knee pain over the past two years. Her pain was mainly over the anterior aspect of the knee and increased during activities like standing, walking, and stair climbing. There was no history of clicking in the knee. Occasionally, the knee would swell and catch. There was no history of pain at rest and night. Past history includes carcinoma of the left breast two years before this presentation for which a left-sided mastectomy was done. There was no history of radiation or chemotherapy following this, but she was started on Tamoxifen for five years. In her last visit with her oncologist, the patient was cleared of metastases. Another significant history includes superficial burns over a large area of the left leg and thigh for which split skin grafting was done three years before.

Upon examination of the knee, the skin graft appeared well integrated over the anterior and lateral aspects of the knee and lower thigh. There was a grade 1 effusion in the knee. The knee was stable for anterior cruciate ligament (ACL), posterior cruciate ligament (PCL), and collaterals, and the range of movement was full and painless. 

**Figure 1:**
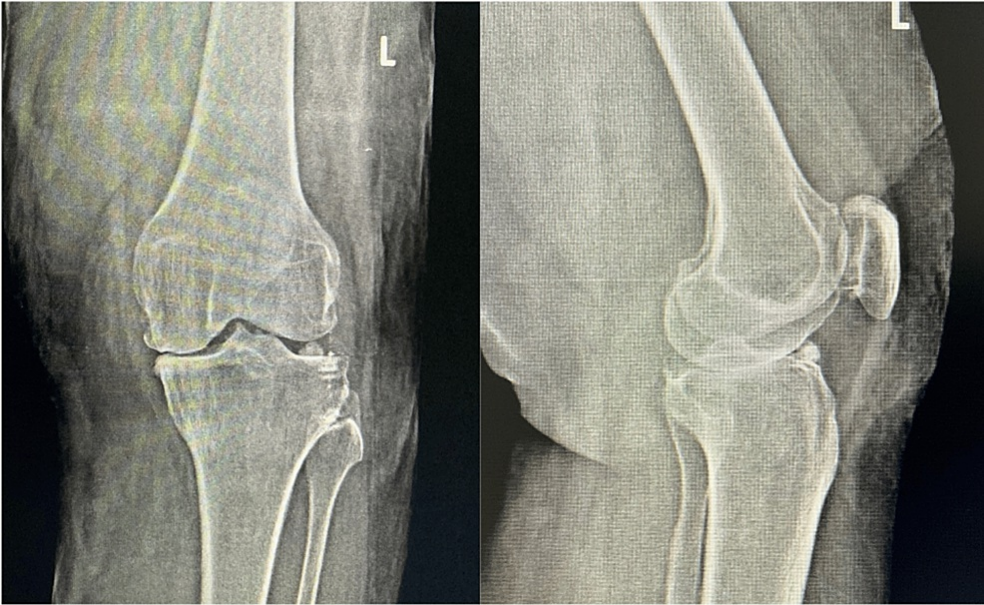
Standing AP and lateral radiographs of the knee revealed a loose body located anteriorly in the lateral compartment, alongside advanced osteoarthritic changes in the medial compartment.

A knee radiograph showed grade 3 Kellgren-Lawrence (KL) osteoarthritic changes in the medial compartment and a loose body in the lateral compartment (Figure 1).

She was offered arthroscopy of the knee and loose body excision and joint replacement after a thorough discussion, and she opted to have arthroscopy. 

**Figure 2:**
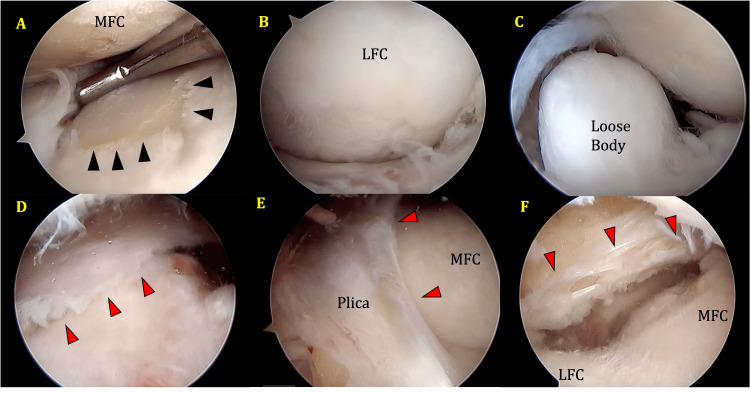
(A) Grade 4 OA changes in the medial compartment: femoral and tibial sides (black arrowhead outlines grade 4 OA changes on tibial surface); (B) LFC showing grade 3 OA changes; (C) loose osteocartilaginous body seen in the intercondylar notch area with LFC in the background; (D) lateral suprapatellar portal view showing plica over the MFC (red arrowheads outline the course of the plica); (E) anterolateral portal view of the plica covering the MFC and extending onto the trochlea; the plica covers most of the MFC; and (F) plica (arrowheads) seen in the infrapatellar region through the lateral suprapatellar portal. MFC and LFC are labeled. Underlying AC defect seen in the MFC under the plica. MFC, medial femoral condyle; LFC, lateral femoral condyle; OA, osteoarthritis

**Figure 3:**
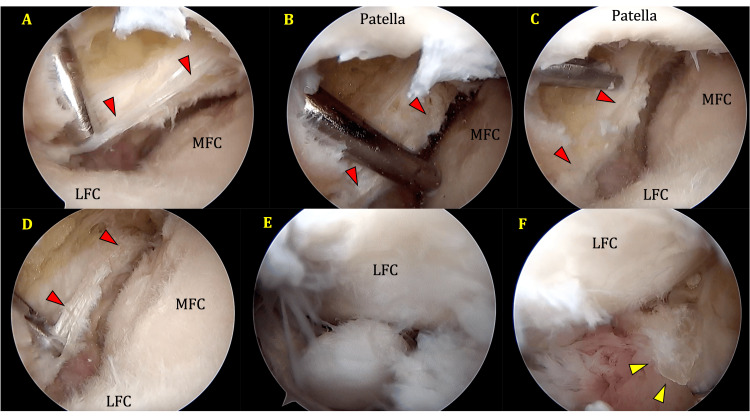
(A) to (D) Lateral suprapatellar portal view of the plica seen in the infrapatellar region (red arrowheads outline the course of the plica in each view); (E) loose body seen in the intercondylar area with LFC in the background; (F) AL portal view after excision of the plica (yellow arrowheads point to the cut surface of the plica). MFC, medial femoral condyle; LFC, lateral femoral condyle

Examination under anesthesia did not reveal any significant findings. Arthroscopy showed grade 4 osteoarthritic changes in the medial compartment on the femoral and tibial sides, a loose body in the lateral compartment as well as a plica. The medial compartment could not be visualized until the plica was cleared. The plica extended as a medial patellar plica (MPP) in the suprapatellar region down to the infrapatellar area as a coronal plane plica. It covered the medial femoral condyle (MFC) like a curtain and unlike the classical MPP whose lateral extent does not cross the level of the lateral margin of the patellar tendon inferiorly, this plica extended well laterally, as the lateral part of a biblike plica. It appeared to be a merged MPP and ICPP. The lateral insertion of this plica was similar to the point described in a previous publication [2]. Examination of the plica was easier using a lateral supra patellar portal (Figures 2F, 3A-3E).

The free margin was thick and fibrosed. The plica was excised, especially in the infrapatellar region, and the loose body was removed.

Postoperatively, the knee was mobilized full weight-bearing. She was asymptomatic for three months postoperatively. Following this, she reported posterior knee and leg pain, which was new and appeared to be arising from her advanced osteoarthritis (OA). She opted for a platelet-rich plasma injection at three months postoperatively. The pain improved following this injection. Her symptoms progressed after six months as she had advanced OA, and a total knee arthroplasty (TKA) was done 11 months after the arthroscopy. At the last follow-up, 17 months after the index surgery and six months after TKA, she was asymptomatic. Her knee mobilized well independently, with a range of movement from 0° to 120°, and she had a stable knee.

## Discussion

The most important finding from this case is that the literature on coronal plane plicae is evolving. The embryological development of the plicae is not clear. Although sagittal plane plicae have been thought to originate from incomplete resolution of septae separating individual compartments of the knee, the origin of coronal plane plicae is not clear [2].

Our case is an elderly lady for whom an arthroscopy was performed for the removal of a loose body. It is not clear whether the symptoms in her were caused by the plica, the degenerative changes, or the loose body. Removal of the loose body and excision of the plica temporarily resolved her symptoms. Dandy [3] thought those complete plicae contracted with age. He found that only 4% of plicae extended two-thirds across the MFC.

It could explain the late presentation in our case if such a large structure contracted over age if it were symptomatic. Arthroscopy is only indicated for symptomatic loose bodies in the presence of degenerative arthritis. Our patient’s symptoms were indicative of a loose body rather than advanced OA. Hence, arthroscopy was offered for the removal of the loose body.

Plicae have been classified into different types based on the completeness of the structure since there is considerable variability in the occurrence and morphology of these structures. Our plica does not fit into any of the described patterns or their variants [1,6,7].

The distal attachment of the MPP given in the literature does not cross lateral to the patellar tendon. Most studies in the literature do not give the distal attachment accurately, but it does not appear to extend laterally beyond the infrapatellar fat pad [8]. In our case, the attachment extends well laterally into the lateral compartment, almost reaching the mid-lateral plane, as described by Kambhampati et al. [2] for the lateral attachment of their reported infrapatellar plica. The component that extends laterally is entirely infrapatellar, as seen in Figures 2-3. Hence, it should be considered similar to the lateral half of the ICPP. The medial half appears to have merged with the MPP and extended over the MFC into the suprapatellar region like the MPP, as depicted in Figures 2-3. It has been observed that the MPP obstructs the placement of the anteromedial portal, and the best view is obtained from a suprapatellar portal. We present the findings from the suprapatellar portal. The lateral part of the plica did not obstruct our regular high anterolateral portal. It seems to be positioned slightly lower than in the earlier case report [2], thus avoiding obstruction of a high anterolateral portal.

This is a distinct structure in the knee that appears to be posterior to the infrapatellar fat pad. There was evidence of grade 3 AC defects in the MFC anteriorly under cover of the plica. It appeared to wrap the MFC during flexion of the knee. Adjacent weight-bearing areas of MFC and MTP in the tibiofemoral articulation have demonstrated grade 4 degenerative changes. Menisci and cruciate ligaments appeared intact.

There is no evidence of concomitant transverse plane or sagittal plane plicae in the presence of this plica. We did not perform an MRI scan in this case as the symptoms were thought to be due to a loose body in a degenerative knee.

The patient had resolved her symptoms after excision of the plica and the loose body. Four weeks after arthroscopic plica excision, the patient received a platelet-rich plasma injection for her degenerative changes, leading to further resolution of her symptoms. It is possible that her symptoms were entirely due to OA and loose body and that this plica was asymptomatic. Resolution of her symptoms following excision indicates a role for the plica in her symptoms. Liu et al. found that all types of MPP caused injuries due to the pressure on the MFC when the knee flexed beyond 50 degrees [4].

**Figure 4:**
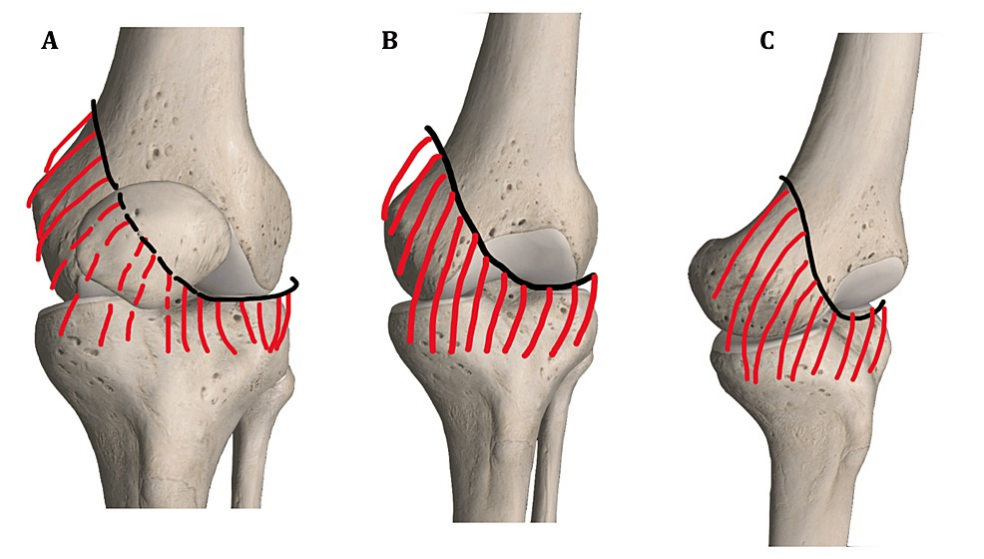
Plica viewed from the (A) front, (B) obliquely, and (C) laterally. The patella has been removed in (B) and (C) to depict the course of the plica. The solid arrow in (B) shows the area of the plica that can potentially restrict patellar eversion during TKA. TKA, total knee arthroplasty

During the TKA of this patient, it was found that the fibrous tissue from the excised plica, particularly the lateral infrapatellar part, restricted the amount of eversion of the patella during the standard medial parapatellar approach. Once this fibrous band was released, patellar eversion and the approach became easier. Hence, in patients with this variant of plica and even after excision, awareness of this possibility and releasing the structure laterally could improve the eversion of the patella and exposure of the knee (solid arrow, Figure 4B).

Eversion of the patella during TKA may also be difficult due to scarring after previous surgeries [9].

## Conclusions

EMCPP is a rare variant of plica noted in an elderly lady. Its role in symptoms is not clear, but it could erode the adjacent MFC. Excision causes the resolution of symptoms.

The plica itself or the resulting fibrotic band after excision could potentially affect patellar tracking and patellar eversion during knee exposures and TKA.
